# Comparison of Equimolar Doses of Mannitol and Hypertonic Saline for the Treatment of Elevated Intracranial Pressure After Traumatic Brain Injury: A Systematic Review and Meta-Analysis: Erratum

**DOI:** 10.1097/01.md.0000489122.14684.45

**Published:** 2016-07-08

In the article “Comparison of Equimolar Doses of Mannitol and Hypertonic Saline for the Treatment of Elevated Intracranial Pressure After Traumatic Brain Injury: A Systematic Review and Meta-Analysis”,^1^ which appeared in Volume 94, Issue 17 of *Medicine*, an incorrect doi was linked to the article. The article has since been corrected online.
